# Dynamic Migration Characteristics of Potassium During Agricultural Waste Combustion and the Mechanism of Combined Chlorine–Sulfur Action

**DOI:** 10.3390/molecules30122495

**Published:** 2025-06-06

**Authors:** Jian Li, Yunlong Zhou, Guochao Zhao, Qixin Yuan

**Affiliations:** 1 School of Energy and Power Engineering, Northeast Electric Power University, Jilin City 132012, China; leejian0519@163.com; 2 Datang Northeast Electric Power Test & Research Institute Co., Ltd., Changchun 130102, China; zgdtzgc@126.com; 3 School of Electrical and Power Engineering, Taiyuan University of Technology, Taiyuan 030024, China; yuanqixin@tyut.edu.cn

**Keywords:** agricultural waste, combustion, potassium, transformation, chlorine–sulfur

## Abstract

Alkali metals in fuel seriously affect the normal operation of generator sets. Using agricultural waste (AW) from a corn field as raw material, the dynamic change of alkali metal K migration and transformation and the effect of competition between chlorine and sulfur on the behavior of AW were studied systematically. The results showed that transformation between different forms of K, especially water-soluble K, occurred. At low temperatures, K remained in the ash in the form of inorganic salt, and high temperature precipitated K and formed insoluble alkali metal compounds. Via FactSage thermodynamic equilibrium calculations, it was confirmed that KCl reacted with SiO_2_ to form a K_2_O·nSiO_2_ molten mixture in combustion. K initially existed in the form of KCl (s) and K_2_SO_4_ (s), high temperature promoted its transformation and decomposition, and it was eventually released as KCl (g). During combustion, Cl was more volatile than K, while S reduced the release of K and Cl through sulfation reaction to reduce the sediment viscosity.

## 1. Introduction

The overutilization and exploitation of mineral resources have caused serious negative impacts on the environment and the Earth. As a clean, environmentally friendly and sustainable form of energy, biomass energy has attracted wide attention in recent years. Its status as a fourth energy source, the large amount of organic matter produced each year, and the fact that it is considered to be carbon dioxide neutral make biomass energy a promising application for future energy systems [1]. The use of biomass energy is of great significance for alleviating the energy shortage and environmental pressure in China.

When biomass is burned as fuel, due to the very high content of alkali metals and alkaline earth metals in the biomass, it not only produces slagging, deposition, and agglomeration in the combustion system but also causes a series of particularly serious problems such as heating surface corrosion [2]. Compounds containing alkali metals can react with silica to form a eutectic mixture with a low melting point that sinters easily at high temperatures [3,4]. It is worth noting that biomass ash has a smooth surface, low porosity [5], and high viscosity and strength. Heat exchangers are key devices that use fuel in the form of heat [6,7]. If too much ash is deposited on the heat exchange surface, the heat transfer rate reduces, and the surface needs to be cleaned frequently [8]. In addition, when the temperature is greater than 500 °C, alkali metals easily combine with chlorine to form volatile alkali chloride [9]. In addition, within the temperature range of 750–950 °C, the potassium salts in biomass ash are highly volatile. The generated alkali chloride reacts with the metal flue, causing high-temperature corrosion of the flue wall [10]. Alkali chloride can react with metal flue, resulting in high-temperature corrosion of the flue wall [10].

Temperature is the key factor affecting the migration of K and Cl. Deng et al. [11] and Meng et al. [12] found through experiments that at high temperatures, more K could enter the flue gas, different forms of K was transformed into each other, and the release of HCl could also increase. Mousavi et al. [13] showed via a numerical prediction model that the content of residual water-soluble K reduced with increasing temperature, and the solubility of K in NH_4_Ac further decreased at higher temperature. Wang et al. [14] studied the dynamic migration and transformation characteristics of K during combustion and found that water-soluble K was the main form affecting the combustion characteristics of straw carbon. Fatehi et al. [15] confirmed through comparative model calculations and experimental measurement that KCl, HCl, and K(g) were the main forms of chlorine and potassium in the presence of chlorine, while K(g) played a leading role in the release of potassium in the absence of chlorine. Fatehi et al. [15] confirmed through comparative model calculations and experimental measurements that in the presence of chlorine, KCl, HCl, and K(g) are the main forms of chlorine and potassium, while in the absence of chlorine, K(g) plays a dominant role in the release process of potassium; K(g) refers to gaseous K(g). The migration and transformation of K is a dynamic behavior in the combustion process, which not only affects the combustion reaction characteristics in real time but also plays an important role in the prevention and control of ash deposition corrosion. In addition, the competition mechanism between chlorine and sulfur also has a non-negligible impact on the process [16]. However, recent research on K and Cl during biomass combustion has mainly focused on composition analysis and the formation process of the solid phase products, and the related mechanism of K and Cl in the gas phase is rarely studied.

The Northeast region of China is rich in crop straw resources, with an annual output of approximately 100 million tons, accounting for 15% to 20% of the national total [17]. However, the main methods for dealing with agricultural waste are incineration and open-air decomposition, which can cause serious air pollution [18]. In addition, the Northeast region has a colder and drier climate than the Southern region and is short of wood resources. In this context, this study selected agricultural waste (AW) from corn fields in Northeast China as the research object. In this paper, the agricultural waste (AW) extracted from a corn field was used as the research object, and the combustion ash sample was obtained at 500–900 °C in a tube furnace combustion system. The microstructure of combustion products and the composition of the melt were determined through a large number of characterization experiments. Using chemical fractionation, ICP–OES, and ion chromatography, the content variation, migration and transformation of different forms of K and Cl during combustion were analyzed. In addition, the thermodynamic equilibrium calculation software FactSage 8.0 was used to simulate the migration of K and Cl during combustion. The distribution and states of K and Cl in the solid phase and gas phase were determined according to the analysis results. The results of this work help to reveal the transformation and migration mechanism of alkali metals and chlorine during biomass combustion.

## 2. Results and Discussion

### 2.1. Combustion Behavior of AW

With the increase in combustion temperature, the ash residue after AW combustion adhered to the surface of the corundum boat, and a bonding phenomenon occurred above 600 °C. This phenomenon was due to the fact that during the combustion process, K in the AW was precipitated through KCl and chemically reacted with silicon oxide on the surface of the material, resulting in ash bonding and agglomeration. When the temperature reached 900 °C, the ash was tightly attached to the corundum boat, producing non-combustible ash. This indicates that the chemical reaction between K in the ash and silicon oxide was more intense under high temperature conditions, resulting in obvious physical and chemical changes in the ash combustion process. The relevant reaction formulas can be found in Equations (1) and (2) [19]:

(1) $$2KCl+Al_{2}O_{3}+2SiO_{2}+H_{2}O\to 2KAlSiO_{4}+2HCl$$

(2) $$2KCl+Al_{2}O_{3}+2SiO_{2}+H_{2}O+O_{2}\to 2KAlSiO_{6}+2HCl$$

**Figure 1:**
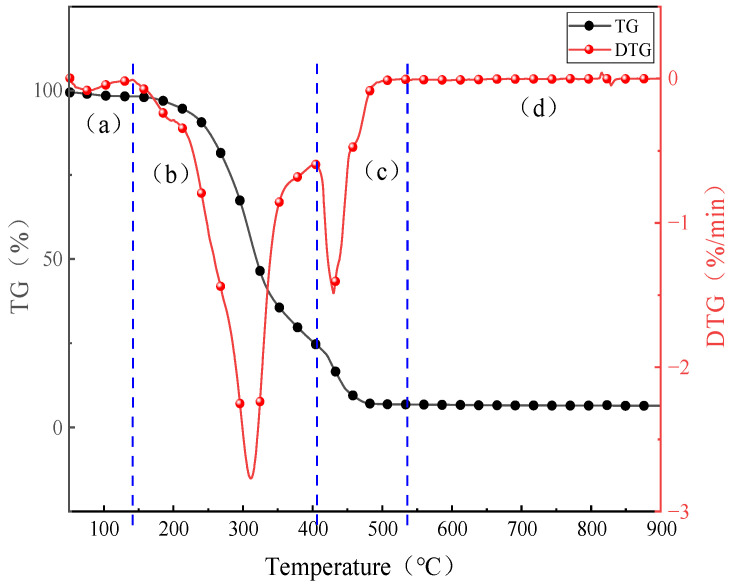
AW combustion TG/DTG curve.(**a**) drying stage; (**b**) devolatilization phase; (**c**) coke combustion; (**d**) burnout stages.

The four stages of the AW combustion process had unique characteristics. It can be clearly seen in Figure 1 that each stage of the AW combustion process had different combustion characteristics.

During the drying stage (a), the evaporation of water in the fuel resulted in a loss of mass. In the devolatilization phase (b), organic and inorganic alkali metals were released into the gas phase through thermal decomposition, thermal conversion, or replacement reactions [20,21,22], and inorganic alkali metals reacted with silica luminate to form mineral salts. In the coke combustion (c) and burnout (d) stages, the ignition combustion of coke caused the particles’ temperature to rise, and the organic alkali metals participated in the oxidation process of the coke to form organic alkali metals in oxidized form and the free alkali metal salts released into the gas phase, and they also reacted with oxygen, HCl, and SO_2_ to form the gas phase alkali metal salts. Organic and inorganic alkali metals can also combine with silicates to form alkali silicates [23,24,25,26].

### 2.2. Migration Behavior of K

#### 2.2.1. Precipitation Characteristics of K

The ICP detection results are shown in Table 1. In the AW, water-soluble K accounted for 87.03% of the total K content, representing the highest proportion. The content of K in ash samples produced by AW combustion at different temperatures varied. In addition, the proportion of insoluble K increased with the increasing temperature, indicating that part of the K was converted from water-soluble and NH_4_Ac-soluble to other forms of K with the increasing combustion temperature. At 900 °C, the proportion of insoluble K increased to about 32.26%, indicating that the K in AW is more inclined to form insoluble compounds at high temperatures.

**Figure 2:**
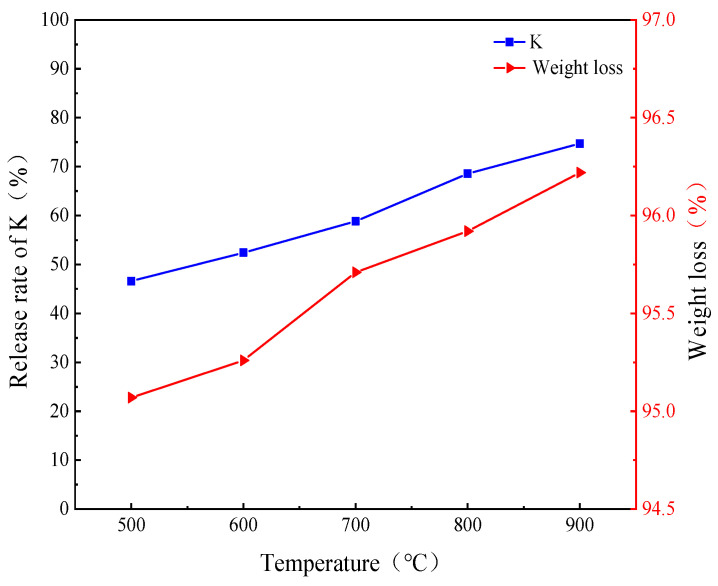
Relationship between combustion weight loss rate and K release rate at different temperatures.

It can be observed in Figure 2 that the combustion weight loss rate of the AW gradually increased with the increase in temperature. The weight loss rate in all the combustion experiments was above 95%, indicating that the AW underwent very serious mass loss and demonstrated a large weight loss ratio during combustion. Further study found that in the case of complete burnout, the rate of increase in the combustion weight loss was slower in the temperature range of 500–600 °C, while the rate of increase was faster in the temperature range of 600–900 °C. The content of alkali metal K in ash samples decreased gradually with the increase in combustion weight loss. This shows that in the early stage of combustion, alkali metal K gradually precipitated during the volatilization analysis stage, and in the later stage of combustion, the thermal instability of organic K led to alkali metal K precipitation. In addition, in the temperature range of 500–900 °C, the content of K gradually decreased due to the increase in temperature, which indicates that the increase in temperature promoted the precipitation of K and entry into the gas phase. These phenomena may be related to the chemical properties of K in biomass, its volatility, and the reaction during combustion [27]. K may be present in different forms in biomass, such as alkali metal salts or organic compounds, leading to differences in its behavior during combustion. In addition, under high temperature conditions, organic matter in biomass can further decompose, resulting in the release of more volatile elements, including K [28,29,30]. These results show that the behavior of alkali metal K is closely related to the weight loss rate during combustion. With the combustion process, the increased weight loss led to an increase in alkali metal K precipitation, and the precipitation rate slowed down at the high-temperature stage.

#### 2.2.2. Migration and Transformation Characteristics of K

**Figure 3:**
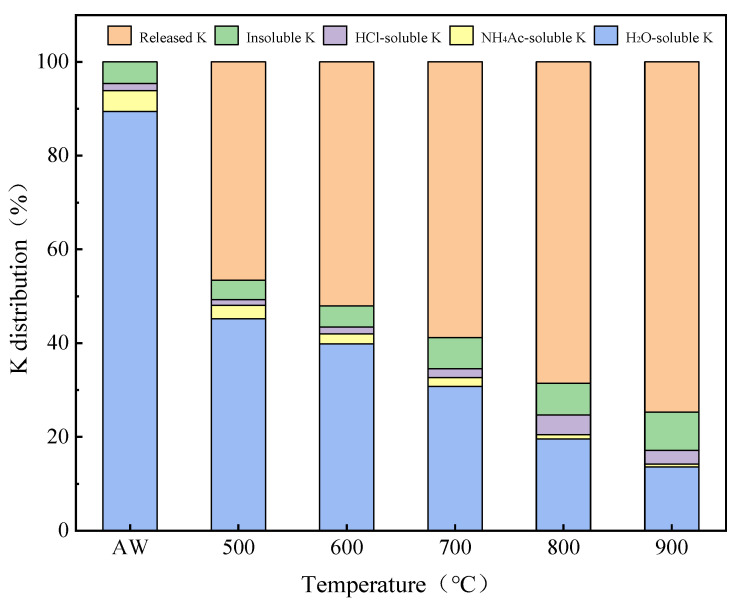
Distribution proportion of K in AW and ash samples after combustion at different temperatures.

The combustion temperature had a significant effect on the release behavior and form of alkali metal K. There was an obvious turning point around 700 °C, when the rate of K entering the gas phase increased significantly, mainly in water-soluble form. Figure 3 shows that with the increase in temperature, especially at 900 °C, the precipitation of K was at its most significant, and the content of K in the slag was at its lowest level. At the same time, the contents of soluble K and insoluble K in HCl increased, while the contents of soluble K and NH_4_Ac-soluble K decreased. This is because in the combustion process, alkali metal K exists in different chemical forms, and a chemical reaction between them transforms these, especially under high temperature conditions; insoluble alkali metal compounds are formed, thereby reducing the release of water-soluble K, and the increase in the soluble K content of HCl may be related to the transformation of water-soluble K and NH_4_Ac-soluble K [28,31,32]. Some scholars believe that at low temperatures, water-soluble K and NH_4_Ac-soluble K can react with silicate to form organic binding K [31]. The presence and transformation of different forms of alkali metal K in the solid phase are affected by temperature. At lower temperatures, K exists in the form of silicate and does not form KCl in the gaseous state. As the temperature rises, K is released in gaseous form such as KCl and (KCl)_2_, and the K content in the solid phase gradually decreases, which indicates that the increase in temperature is conducive to the transition of gas–solid equilibrium to the formation of the gas phase, resulting in more K precipitation in the form of gas [33].

At low temperatures, when AW is burned, alkali metal K remains in the ash in the form of inorganic salt. This is because at low temperatures, the reaction rate is slow, the gas phase components are less, and the ash residue is greater, which limits the evaporation and release of K. This has an effect on the combustion process. Therefore, in the combustion of AW with high volatile content at low temperature, K remains in the ash in the form of inorganic salt.

#### 2.2.3. Change Rule of Minerals in Ash with Temperature Rise

**Figure 4:**
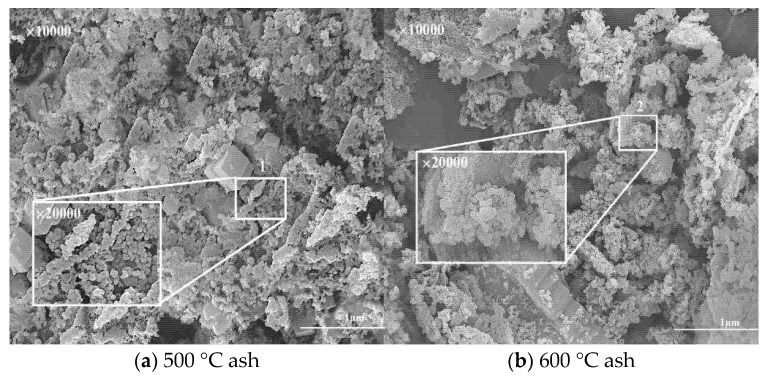

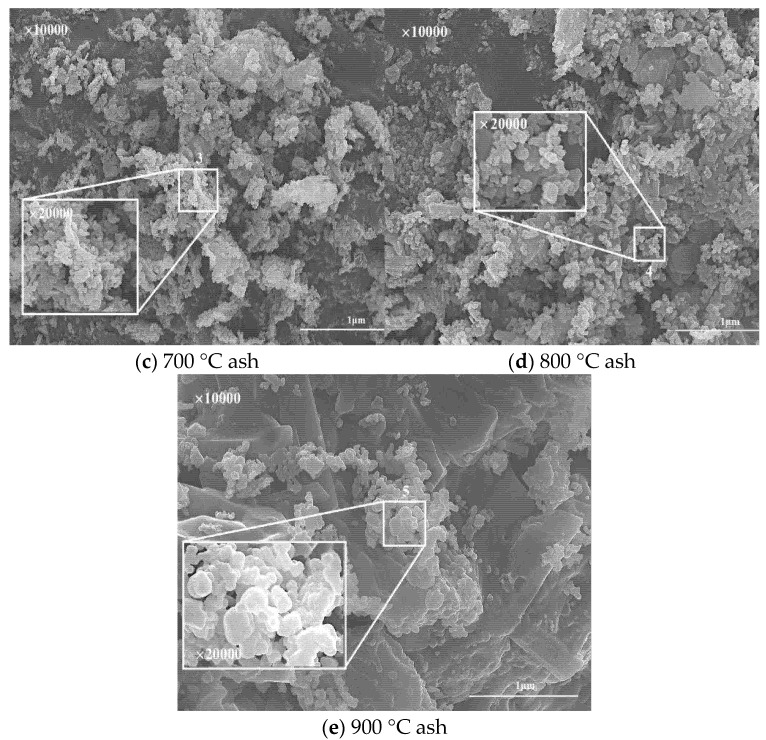
SEM scan image of AW gray sample. (1–5) The magnified point position.

SEM was used to study the morphological changes and existent forms of AW ash samples after combustion at different temperatures, as shown in Figure 4.

The results show that at lower temperatures, the ash sample still retained the fibrous structure, while at higher temperatures, the ash sample became blocky and the surface holes were uneven, and the melt is obviously attached to the surface. This may be because at high temperatures, the organic component of AW is completely decomposed during combustion, resulting in the ash losing its fibrous structure, while the mineral component melts to form a molten mixture at high temperatures [2,34]. At 900 °C, the grain structure of the AW ash sample was angular, and a certain amount of molten mixture adhered to the surface. The main component may have been SiO_2_, which indicates that during the AW combustion process, alkali metal K existed in the ash in the form of inorganic salt, and with the increase in temperature, SiO_2_ was precipitated from the organic matter and formed an independent granular structure. This is consistent with the results of chemical composition analysis of AW ash samples in previous studies and further supports the inference that SiO_2_ was the main component.

A representative region was selected from the scanning images of combustion products at various temperatures for EDS spectrum analysis, and the results are shown in Table 2.

It was found that the main components of the sample surface were C, O, Si, K and Cl. In addition, small amounts of Mg, Ca, Al and other elements were detected. The main elements on the surface of the residue after combustion were C and O, and the mass percentage of C was 1.42% under the combustion conditions at 500 °C. These results show that these elements are relatively stable during combustion and can be stably retained in ash samples after oxidation reactions.

**Figure 5:**
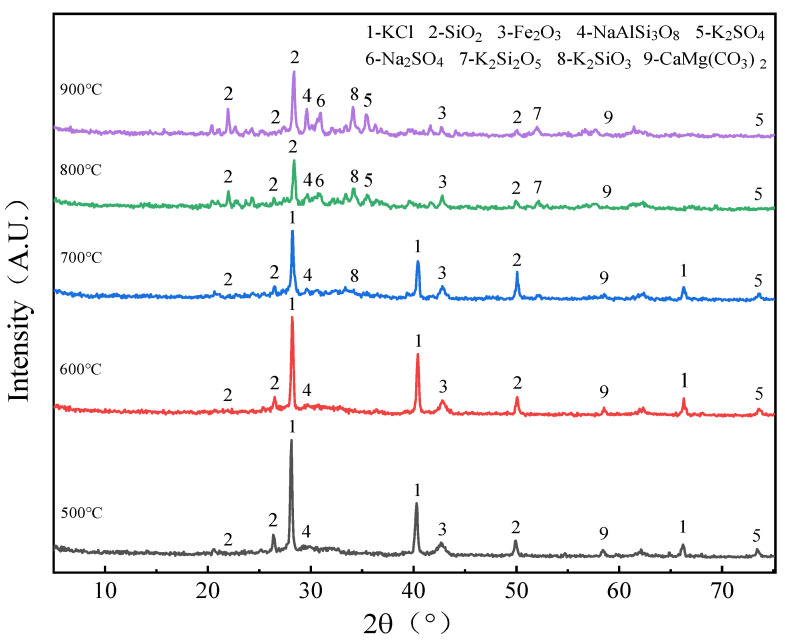
XRD scan results of AW combustion ash.

The XRD test results are shown in Figure 5.

With the increase in combustion temperature, the intensity of the SiO_2_ peaks increased, but the peaks of all KCl crystalline phases were significantly weakened, which indicates that when the temperature gradually increased, K in the AW combustion residue evaporated into the gas phase, and the migration form of the K was mainly KCl(g). In addition, in the residues burned at 800 °C and 900 °C, the XRD pattern showed more amorphous substances, which were not diffracted by X-rays [3] and thus formed a “mountain”-shaped baseline. As shown in Figure 5, it was found that these amorphous substances were mainly fused substances adhering to the surface of the granular residue, which was a mixture of K_2_O·nSiO_2_ generated by the reaction of KCl and SiO_2_.

Oxides are the main components of fuel ash slagging/scaling, including alkali and alkaline earth metals, silicon, chlorine, and sulfur [10]. As can be seen from Table 2, the contents of K_2_O and Cl gradually decreased due to the gradual increase in temperature, and at a high temperature above 800 °C, elements such as K and Cl in ash were hardly detected in the main phase. The content of SiO_2_ in the residue increased significantly with the increase in temperature. At 800 °C, the content of SiO_2_ reached 35.5%, which indicates that at high temperatures, SiO_2_ became the main component of the sample. At the same time, some stable elements such as Fe, Al, and Mg underwent oxidation reactions to generate corresponding oxides, and SiO_2_, MgO and other components became the main components in the ash [35].

#### 2.2.4. Migration and Transformation of K During Combustion

According to the analysis of the AW combustion experiment results, with the increase in temperature, KCl continued to be released into the gas phase until it was completely released. At the same time, a small amount of K was converted to K_2_CO_3_ and subsequently decomposed and released to form the insoluble silicate K_2_SiO_3_. KCl cannot be directly converted to K_2_SiO_3_, but first migrates to the gas phase and then reacts with SiO_2_ to produce K_2_SiO_3_. In the combustion process, the main volatile salts were KCl and K_2_CO_3_, and the content of K_2_SO_4_ was less. This shows that in the temperature range of 500–700 °C, the main compounds of K and Cl in the AW ash were mainly KCl, and there were also some trace compounds such as K_2_SO_4_. The reaction formula for K precipitation caused by sublimation, evaporation, or decomposition is as follows [28,36,37]:

(3) KCl(s,l)→KCl(g)

(4) $$2KCl(s,l)\to (KCl{)}_{2}(g)$$

(5) $$K_{2}SO_{4}(s,l)\to K_{2}SO_{4}(g)$$

(6) $$K_{2}CO_{3}(s,l)+H_{2}O\to 2KOH(g)+CO_{2}(g)$$

(7) $$K_{2}CO_{3}+SiO_{2}\to K_{2}SiO_{3}+CO_{2}(g)$$

In addition, the decomposition of K_2_CO_3_ occurs in the absence of water, as follows, but does not cause the precipitation of K:

(8) $$K_{2}CO_{3}(s,l)\to K_{2}O(s,l)+CO_{2}(g)$$

The resulting K_2_O can combine with SiO_2_, and there is a small amount of decomposition to produce K(g).

(9) $$K_{2}O\cdot SiO_{2}+Al_{2}O_{3}\cdot 2SiO_{2}\to K_{2}O\cdot Al_{2}O_{3}\cdot 2SiO_{2}+SiO_{2}$$

(10) $$2KCl+SiO_{2}+H_{2}O(g)\to K_{2}SiO_{3}+2HCl(g)$$

(11) $$4KCl+2H_{2}O(g)+O_{2}+2SO_{2}(g)\to 2K_{2}SO_{4}+4HCl(g)$$

In summary, K during the AW combustion was present in the gas phase release of KCl and solid phase formation of K_2_SiO_3_. During the devolatilization stage, KCl was quickly released into the gas phase and a small amount of organic K was released. Unreleased organic K existed as a residue. As the temperature increased, KCl continued to be released into the gas phase until it was completely released. At the same time, a small amount of K was converted to K_2_CO_3_ and decomposed to release, forming the insoluble silicate K_2_SiO_3_. In the combustion process, the main volatile salts were KCl and K_2_CO_3_, and the content of K_2_SO_4_ was less [38,39]. The K and Cl in the AW ash are mainly released into the gas phase in the form of KCl, accompanied by the precipitation of HCl. At higher temperatures, the main components of the ash were still SiO_2_, MgO, etc., the Cl content in the ash decreased, the K content changed, and the precipitation types of K and Cl were still mainly K-containing compounds.

### 2.3. Research on the Dynamic Migration Law of Potassium Using FactSage

**Figure 6:**
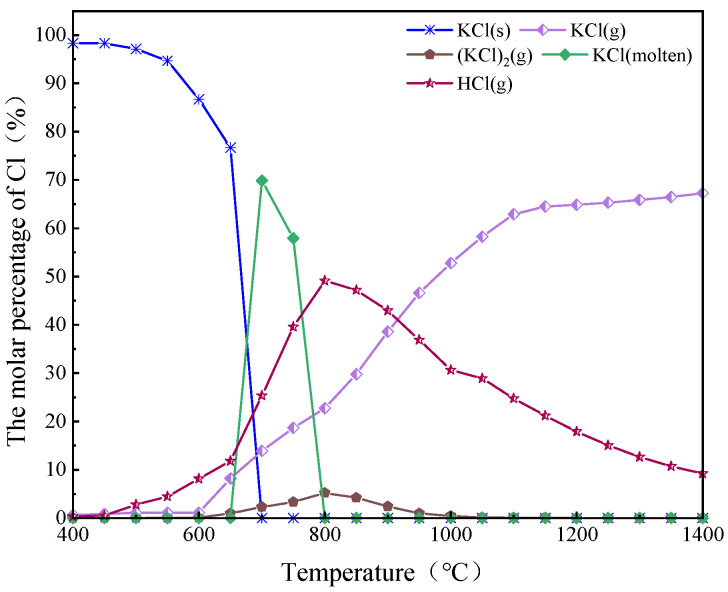
Heat balance distribution of main Cl-containing compounds during combustion.

Figure 6 shows that when the temperature was lower than 700 °C, Cl in the AW mainly existed in the form of KCl(s).

The release of K was closely related to the content of Cl [40]. In the range of 500–800 °C, the content of HCl(g) gradually increased, reaching a maximum at about 800 °C, while the content of solid potassium chloride and molten potassium chloride dropped to zero, meaning that HCl(g) was transformed from molten KCl(s) and molten KCl. When the temperature was higher than 800 °C, the content of HCl(g) gradually decreased, the content of KCl(g) gradually increased, and a small amount of (KCl)_2_(g) was generated, which indicates that KCl occurs in gas conversion at high temperatures. These results indicate that temperature has a significant effect on the conversion behavior of chloride during combustion.

**Figure 7:**
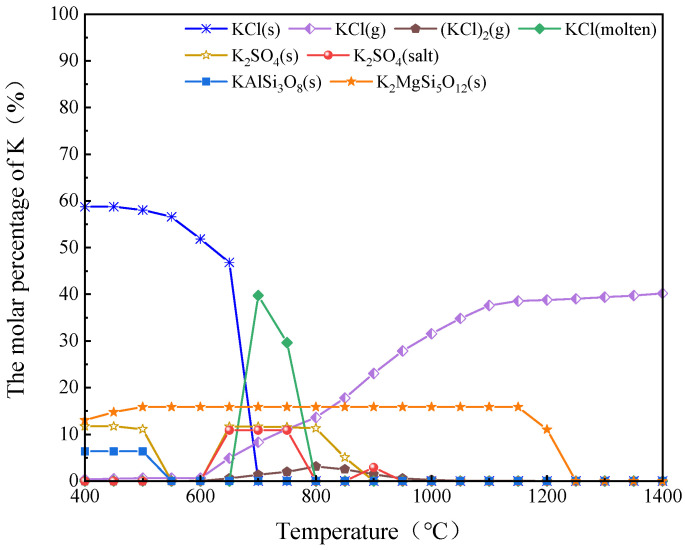
Heat balance distribution of main K-containing compounds during combustion.

Figure 7 shows the equilibrium of K in the AW combustion process, where g, s, and molten represents gaseous, solid, and molten states. The change of phase state and existent form of K are closely related to the occurrence of ash-related problems. During the thermal conversion process, K first entered the gas phase as organic (below 500 °C) or inorganic (above 500 °C) [41]. With the progress of heating and volatilization, water-soluble K^+^ can combine with anions with low thermal stability, form precipitates in the form of inorganic salts, or exchange ions with functional groups of carbon matrix. In the range of 400–650 °C, K existed in solid forms such as KCl(s), K_2_SO_4_(s), KAlSi_3_O_8_(s), K_2_MgSi_5_O_12_(s) during combustion, which is consistent with relevant studies [37,42]. When the temperature reached 650 °C, a large amount of KCl(s) was transformed into molten KCl and gaseous KCl.

It can also be seen in Figure 7 that K_2_SO_4_(s) has better stability than KCl(s), but K_2_SO_4_ also began to transform into a molten state at a high temperature of 650–800 °C. In the temperature range of 800–1000 °C, the decomposition rate of K_2_SO_4_(s) accelerated and it gradually transformed into gaseous products. Such gaseous products can be discharged with the flue gas during combustion due to the following reactions [41,43]:

(12) $$2K_{2}SO_{4}+2Al_{2}O_{3}\cdot 2SiO_{2}\to 4KAlSiO_{4}+2SO_{2}(g)+O_{2}(g)$$

The migration behavior of K and its related elements during AW combustion can be summarized as follows. First, the increase in temperature led to a decrease in the content of KCl(s) and K_2_SO_4_(s) while HCl(g) was precipitated. When the temperature reached 700 °C, KCl(s) completely transformed into the form of KCl(g). (KCl)_2_(g) and molten KCl(g) were present; (KCl)_2_(g) formed around 650 °C, reaching its highest concentration at 800 °C. With the increase in temperature, the content of KCl(g) increased, because the KCl vapor pressure increased.

### 2.4. Influence of Competition Mechanism Between Chlorine and Sulfur on Migration and Transformation of Potassium

#### 2.4.1. Effects of Chlorine on Potassium Release and Transformation

The migration process of alkali metals is significantly controlled by chlorine content, and the release of chlorine-related substances during combustion is the main cause of active corrosion in biomass combustion [44]. Chlorine exists in the form of KCl in biomass fuel, and gaseous compounds are released after the temperature rises, mainly in the form of KCl. At low temperatures, the release of chlorine is higher, and the vapor pressure of KCl is negligible. The release mechanism of chlorine includes HCl precipitation and alkali metal chloride volatilization, among which the possible mechanisms of HCl precipitation includes direct precipitation from fuel and re-capture in coke through secondary reaction with existing metals [45]. The reaction is as follows:

(13) R−COOH(s)+MCl(s)→R−COOM(s)+HCl(g)

where M is an alkali metal and R is an organic functional group.

(14) $$2MCl(g,l)+H_{2}O(g)+xY(s)↔M_{2}O\cdot xY(s,l)+2HCl(g)$$

where Y(s) indicates a pure silicon phase.

**Figure 8:**
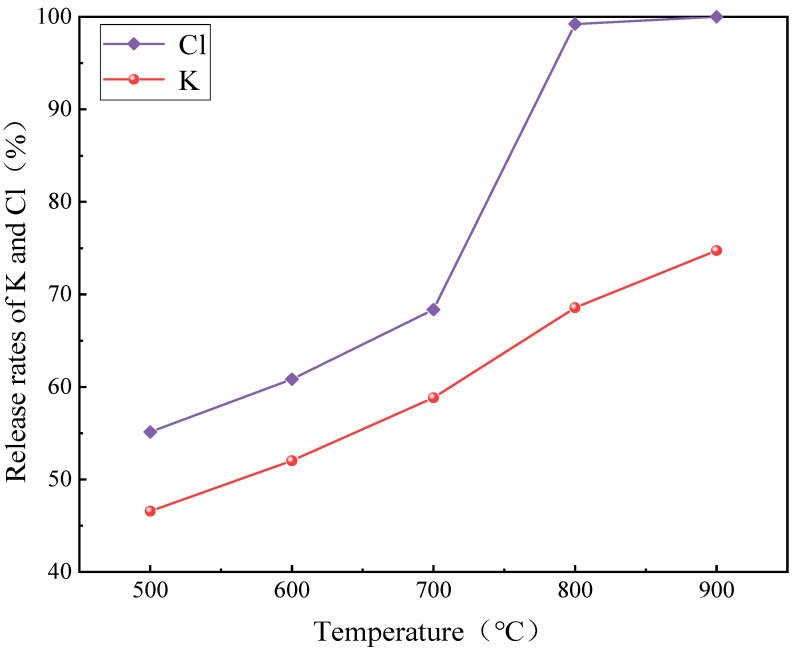
Release rates of K and Cl at different temperatures.

Under high temperature conditions, the chloride evaporates from the solid rather than being released by Reaction (22). At initial low temperature, Cl release is dominated by Reaction (21), while sublimation of alkali metal chloride mainly promotes Cl release at high temperature [38]. Temperature is an important parameter, which has a significant effect on the precipitation behavior of Cl and K. Figure 8 shows that at temperatures below 700 °C, the AW ash released over 50% of its Cl. In the range of 500–600 °C, the release rates of Cl and K increased. The Cl and K precipitated from the AW ash were in the form of gaseous compounds, mainly forming KCl, and K_2_SO_4_ and other substances were also detected in the spectrum. This shows that at low temperatures, Cl and K transformed and precipitated each other in the form of gaseous compounds in the AW ash. In the temperature range of 600–700 °C, the content of Cl and K decreased significantly, mainly precipitating in the form of KCl. In the temperature range of 700–800 °C, the contents of Cl and K decreased significantly, and the decrease in K was greater than that of Cl. At 800–900 °C, the release rate of Cl was basically unchanged, and the combustion was complete when the temperature was raised to 900 °C, which is consistent with relevant studies [46].

#### 2.4.2. Joint Competition Mechanism of Chlorine, Sulfur, and Potassium

In previous studies, it was found that the interaction between biomass and coal inhibited the release of K, so that its release rate did not change linearly with the biomass mixing ratio. Moreover, the higher the biomass ratio, the earlier the peak concentration of alkali metal compounds appeared, and the greater the peak concentration of K [1,47]. Therefore, by changing the blending ratio of AW in Fujian bituminous coal (25 wt.%, 50 wt.%, 75 wt.%) and utilizing the joint competition relationship of S, Cl, and K, this paper further reveals the influence of Cl on the release and transformation of K. The chemical analysis results of different fuel combinations are shown in Table 3.

**Figure 9:**
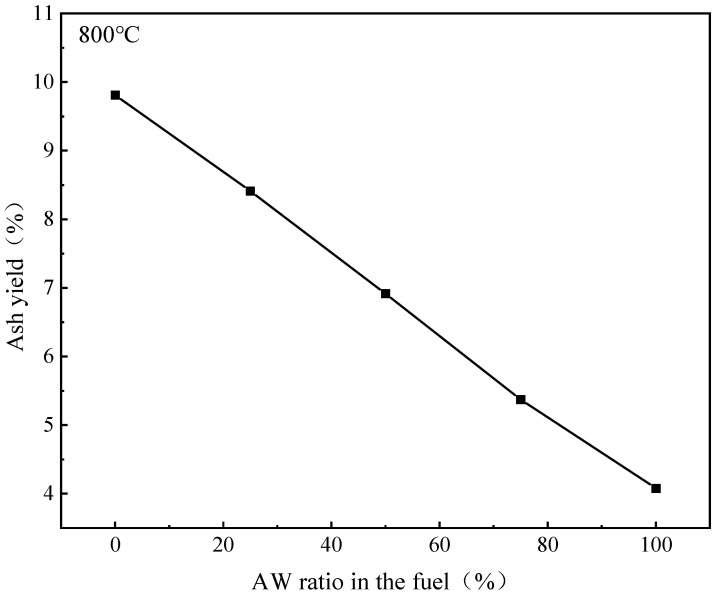
Ash yield at different mixing ratios at 800 °C.

By changing the AW blending ratio and conducting experiments and calculations, the relationship curve between ash yield and AW blending ratio was obtained, as shown in Figure 9.

The results show that the ash yield does not linearly decrease with the increase in the AW blending ratio, because basic aluminosilicates are formed when coal and mineral elements in AW coexist, and more compounds remain in the ash [47].

**Figure 10:**
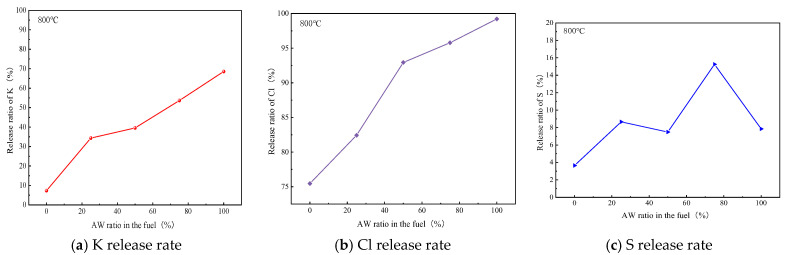
Release rates of (**a**) K, (**b**) Cl, and (**c**) S with different ratios of AW at 800 °C.

Through experimental and theoretical calculations, the relationship between the release rate of K in the mixed fuel and the mixing ratio of the AW is shown in Figure 10. As can be seen from the results in Figure 10, at 800 °C, the release rate of K from pure AW was higher than that from mixed fuel. When the content of AW was 25–50%, the release rate of K increased slowly and it increased sharply when the content of AW exceeded 50%, because the increase in Cl content promoted the precipitation of K [48]. In addition, compared with K and S, the release rate of Cl was the highest, and Cl has a strong effect on the volatilization of alkali metals in the gas phase. The gas phase release rate of S showed an M-type change, and the maximum value was about 15.25% when the AW content was 75%. The release rate of Cl is faster than that of S during combustion. During combustion, HCl and alkali metal chloride are released, and the latter may be completely oxidized. Therefore, in the presence of O_2_ and SO_2_, Cl_2_ and alkali metal sulfate are formed through sulfation reaction. As shown in the equation, the melting point of alkaline sulfate is higher than that of alkaline chloride [16].

(15) $$2MCl\left(g\right)+SO_{2}\left(g\right)+O_{2}\left(g\right)\to M_{2}SO_{4}\left(g\right)+Cl_{2}\left(g\right)$$

Because KCl in AW reacts with Si in coal to release HCl, the amount of HCl released in mixed combustion is more than that in pure AW combustion. In addition, with the increase of AW content, K or Ca in AW reacts with S in coal, and SO_2_ emission decreases. The competition mechanism of S and Cl for alkali metal migration and transformation in the solid phase is as follows [49]:

(16) $$2MCl+xAl_{2}O_{3}+2ySiO_{2}+H_{2}O\to 2MAl_{x}Si_{y}O_{\left(1.5x+2y+0.5\right)}+2HCl$$

(17) $$M_{2}SO_{4}+xAl_{2}O_{3}+ySiO_{2}\to 2MAl_{x}SiO_{\left(1.5x+2y+1\right)}+SO_{2}$$

(18) $$M_{2}SO_{4}+nSiO_{2}\to M_{2}O\cdot nSiO_{2}+SO_{2}+0.5O_{2}$$

where M stands for alkali metals K and Na, and n = 1,2,4.

**Figure 11:**
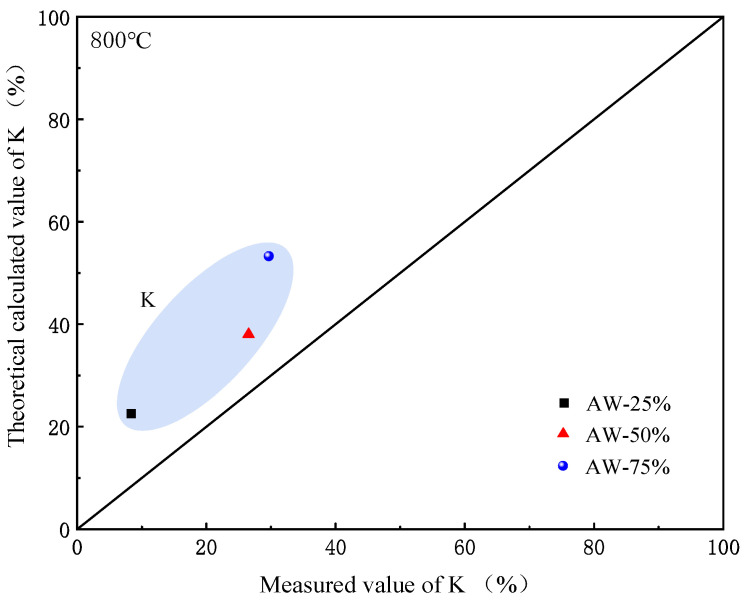
Relationship between the measured value of K release rate and the theoretical calculated value under different AW ratios at 800 °C.

In the co-firing process of AW and coal, the release of K is affected by physical dilution and chemical interaction. In order to distinguish these effects, the theoretical calculated value was obtained based on the linear combination of the measured value of pure AW and pure coal at 800 °C. Figure 11 shows the relationship between the measured value of K and the theoretical calculated value. The results show that regardless of AW ratio, the calculated K release is higher than the actual combined combustion, which indicates that the K release can be inhibited by the interaction between AW and coal. On the one hand, the minerals in the coal can offset the release of KCl [50], and on the other hand, the alkaline substances in the straw react with the S in the coal, thereby reducing the release of K and Cl to reduce the viscosity of the sediment [51]. Therefore, mixed firing can change the chemical reaction path of the fuel, thus affecting the release behavior of metal elements in the fuel. The K in the high-concentration AW bottom ash is captured mainly in the form of K_2_SO_4_, while a small amount of KAlSiO_4_ is produced [52]. When the content of AW was 25 wt.%, 50 wt.%, and 75 wt.%, the release of K was reduced by 63.11%, 30.03%, and 44.25%, respectively. In general, the release rate of Cl is faster than that of S during combustion, and the addition of sulfur-containing compounds can inhibit the release of Cl, which is consistent with relevant research content [16,51,53]. As a result, a high degree of alkali metal sulphation reduces ash accumulation and corrosion problems caused by chlorides.

## 3. Materials and Methods

### 3.1. Raw Material Preparation

The naturally dried agricultural waste (AW) samples were collected from rural areas in Northeast China. They were mainly composed of stalk rind, stalk leaf, and stalk pith of corn stalks. After manual separation, the samples were prepared in accordance with the Chinese national standard GB/T 28730–2012 [54]. Through crushing and screening, powdered samples with particle sizes less than 200 µm were selected. Agricultural waste (AW) was collected from corn fields in Northeast China. The experimental samples were prepared according to the Chinese national standard GB/T 28730–2012, and powder samples with a particle size less than 200 µm were selected through crushing and screening. The samples were dried overnight at 105 °C, after which they were placed in sealed glass bottles for further analysis. The results of industrial analysis (GB/T28731–2012) [55] and elemental analysis (GB/T476–2001) [56] are shown in Table 4. H, O, N, S are elements. AW is fly ash.

### 3.2. Ash Sample Preparation and Properties

**Figure 12:**
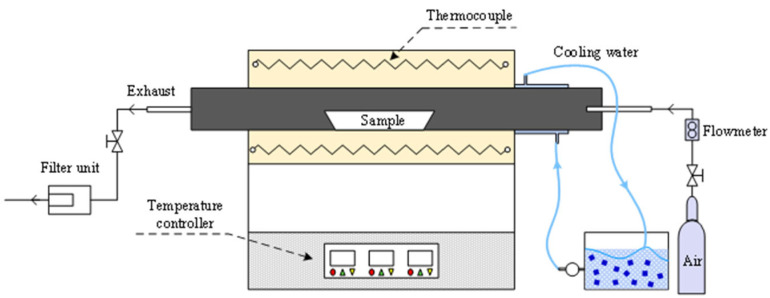
Tube furnace combustion system.

A tubular furnace (OTL 1200, Beijing, China) was used in the experiment, as shown in Figure 12.

AW samples were placed in a corundum boat with a sample mass of no more than 0.1 g per square centimeter. In the experiment, 2 g samples were used and the standard air flow rate was 2 L/min. The combustion temperatures were set at 500 °C, 600 °C, 700 °C, 800 °C, and 900 °C, and the heating rate was 20 °C/min. The experimental steps included (1) setting the temperature parameters; (2) placing the sample boat in the water cooling section of the furnace; (3) fixing and sealing one end of the sample, using the mass flow meter to control the gas flow; (4) heating to the final temperature with continued heating for 5 min, pushing the sample boat into the constant temperature heating zone and keeping the temperature at the specified temperature for 1 h; (5) closing the air valve, quickly moving the sample boat to the water cooling zone and performing rapid cooling.

An X-ray fluorescence spectrometer (XRF, S8 TIGER X, Hamburg, Germany) was used to analyze the chemical components of the ash samples, and the results are shown in Table 5.

### 3.3. Analytical Methods

#### 3.3.1. Chemical Fractionation Analysis

**Figure 13:**
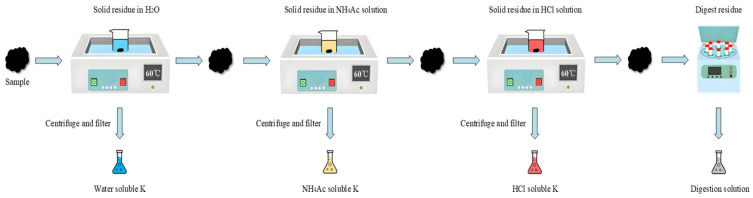
Separation Process of K for Different Existence Forms.

Chemical fractionation is a method commonly used to determine K content in solid samples [54]. The specific operational steps are shown in Figure 13.

The samples were soaked with ultra-pure water, 1 mol/L NH_4_Ac, and 1 mol/L HCl, respectively. The ratio of sample to soaking solution was 1:100 (1 g sample corresponds to 100 mL solution), and the samples were stirred in a water bath at 60 °C for 24 h. After centrifugation at a rotational speed of 3000× g r/min, the supernatant was filtered for subsequent detection. The filter residue was digested with a mixture of 7 mL HNO_3_, 3 mL H_2_O_2_, and 2 mL HF, and the liquid product was obtained at a constant volume [57].

The content of K in the obtained solution was measured by inductively coupled plasma atomic emission spectrometry (ICP-OES) (Agilent 5110, Bellevue, WA, USA). This method can reveal various forms of K in solids, including water solubility, NH_4_Ac solubility, acid solubility, and insoluble residue [58].

#### 3.3.2. Chlorine Content Analysis

The content of Cl in ash samples was quantitatively detected by ion chromatography (IC) (Metrohm 883, Bern, Switzerland).

#### 3.3.3. Thermogravimetric Analysis

The migration of K and Cl showed three different characteristic stages, corresponding to the devolatilization stage of combustion, the coke combustion stage, and the burnout stage [59], which were observed and analyzed using a synchronous thermogravimetric analyzer (TGA-DSC1, 1100LF, Bern, Switzerland). A 9 ± 0.1 mg sample was placed in a crucible and heated from 50 °C to 900 °C in air (flow rate: 50 mL/min) at a heating rate of 20 °C/min. Each experiment was repeated twice, and the relative error of the experimental data was less than 5%.

#### 3.3.4. Scanning Electron Microscope–Energy Dispersive X-Ray Analysis

The surface morphology, element distribution, and precipitation of AW combustion products at different temperatures were studied by scanning electron microscopy (SEM, JSM-7610F, Tokyo, Japan) and energy dispersion spectrometry (EDS, X-Max 80, Abingdon, UK) at 15 KV experimental voltage. To improve the quality of the analysis, all samples were coated with a thin layer of Au-Pd to ensure surface conductivity.

#### 3.3.5. X-Ray Powder Diffraction Analysis

An X-ray diffractometer (XRD, TD-3500, Beijing, China) was used to measure and analyze the crystal structure of the combustion products at different temperatures. The voltage and current were 30 kV and 20 mA, respectively. The diffraction angle range was 5–75°, the scanning speed was set to 2°/min, and the measurement step was 0.068.

### 3.4. Calculation Method

#### 3.4.1. Potassium Content and Release Rate

The K content and ash yield of different samples were different; the ash sample mass of the AW was weighed after combustion at different temperatures to calculate the ash yield. The calculation is shown in Equation (19):

(19) $$\sigma =\frac{m_{h}}{m_{y}}\times 100\%$$

where *σ* is ash yield (%), $m_{h}$ is the mass of ash after AW combustion (g), $m_{y}$ is the mass of AW (g).

The weight loss rate can be calculated from the ash yield, which is calculated via Equation (20):

(20) ω=1−σ

where ω is the weight loss rate (%), *σ* is the ash yield (%).

The concentration of K in solution measured by ICP-OES was converted from Formula (21) to determine the content of K in a 1 g sample.

(21) $$m_{A}=\frac{C_{A}\times V}{m_{S}}$$

where $m_{A}$ is the K content in the sample added during digestion or chemical fractionation (mg/g), $C_{A}$ is the concentration of K in different occurrence forms determined by ICP (mg/L), V is the volume of the solution at constant volume (L), and $m_{S}$ is the mass of the sample added during digestion or chemical fractionation (g).

In addition, after chemical step-by-step extraction, in order to facilitate the study of the conversion between different forms of K occurrence, the results of the forms of K occurrence were converted to the K content contained in the initial raw material. The calculation is shown in Equation (22):

(22) $$M_{A}=m_{A}\times \sigma$$

where $M_{A}$ is the content of K in different forms of occurrence in the initial raw material (mg/g), $m_{A}$ is the content of K in the sample added during digestion or chemical fractionation (mg/g), and $m_{AM_{0}}$ is the ash yield (%).

The release rate of K was calculated via Equation (23):

(23) $$\beta_{K}=\left(1-\frac{m_{AM}\times \sigma }{m_{AM_{0}}}\right)\times 100\%$$

where $\beta_{K}$ is the K release rate (%), σ is the ash yield (%), $m_{AM}$ is the K content in the ash of the sample after combustion (mg/g), $m_{AM_{0}}$ is the K content in the initial sample (mg/g).

#### 3.4.2. Chlorine Release Rate

The release rate of Cl was calculated via Equation (24):

(24) $$\beta_{Cl}=\left(1-\frac{m_{ClM}\times \sigma }{m_{ClM_{0}}}\right)\times 100\%$$

where $\beta_{Cl}$ is the Cl release rate (%), σ is the ash yield (%), $m_{ClM}$ is the Cl content in the ash of the sample after combustion (mg/g), $m_{ClM_{0}}$ is the Cl content in the initial sample (mg/g).

#### 3.4.3. Thermodynamic Equilibrium Calculations

The Equilib module in FactSage was used to calculate the state of K and Cl when they reached chemical equilibrium under certain conditions and to analyze the migration and transformation mechanism. The molar content of AW elements selected for calculation is shown in Table 6. The combustion process of AW was simulated at 21% oxygen concentration. The temperature range was between 400–1400 °C, the temperature step was 50 °C, and the air excess coefficient λ = 1.2. The actual oxygen consumption was calculated via Equations (25) and (26), and the actual nitrogen consumption was calculated via Equations (27).

(25) $$V_{O_{2}}^{0}=1.866\frac{C_{d}}{100}+5.56\frac{H_{d}}{100}+0.7\frac{S_{d}}{100}-0.7\frac{O_{d}}{100}$$

(26) $$V_{O_{2}}=\lambda V_{O_{2}}^{0}$$

(27) $$V_{N_{2}}=\frac{0.79}{0.21}V_{O_{2}}$$

where $V_{O_{2}}^{0}$ is the theoretical oxygen consumption (m^3^/kg), $V_{O_{2}}$ is the actual oxygen consumption (m^3^/kg), and $V_{N_{2}}$ is the actual nitrogen consumption (m^3^/kg).

## 4. Conclusions

During AW combustion, conversion between different forms of K can occur, especially the conversion of water-soluble K to other forms of K. At low temperatures, alkali metal K remains in the ash in the form of inorganic salt. High temperature conditions can cause K to precipitate and enter the gas phase, and K is more inclined to form insoluble alkali metal compounds. As the temperature gradually increases, K in AW combustion residue evaporates into the gas phase; the migration form of K is mainly KCl(g), and the main component in the residue is SiO_2_. The main migration path of K during AW combustion is gas phase release of KCl and solid phase formation of K_2_SiO_3_, in which the main volatile salts are KCl and K_2_CO_3_, and the content of K_2_SO_4_ is relatively small. The FactSage simulation results showed that the content of KCl(s) and K_2_SO_4_(s) decreased with the increase in temperature, while HCl(g) precipitated. When the temperature reached 700 °C, KCl(s) was transformed into KCl(g), (KCl)_2_(g) and molten KCl. As the temperature continued to rise, (KCl)_2_(g) and molten KCl disappeared and the content of KCl(g) increased. During combustion, Cl is more volatile than K, while S can reduce the release of K and Cl through sulfation reaction to reduce sediment viscosity. The melting point of alkaline sulfate is higher than that of alkaline chloride, which is conducive to reducing ash accumulation and corrosion problems caused by alkali metal chloride.

## Figures and Tables

**Table 1:** K content of AW in different forms.

K content of AW in Different Forms (mg/g)	AW	500 °C	600 °C	700 °C	800 °C	900 °C
Water solubility	19.32	10.04	8.84	6.84	4.34	3.02
NH_4_Ac solubility	0.96	0.64	0.48	0.41	0.21	0.12
HCl solubility	0.34	0.27	0.32	0.43	0.93	0.65
Insolubility	0.99	0.92	1.01	1.47	1.50	1.81
Sum total	22.20	11.86	10.65	9.14	6.98	5.61

**Table 2:** Content of elements on AW surface after combustion at different temperatures.

Element	Surface Element Content of Combustion Ash Sample (wt.%)				
	1	2	3	4	5
C	1.42	0.53	0.22	0.14	0
O	0.39	0.52	0.48	0.40	0.97
Si	0.02	0.08	0.06	0.16	0.45
K	0.14	0.10	0.11	0.04	0.17
Cl	0.12	0.06	0.07	0	0
Mg	0.04	0.12	0.11	0.13	0.23
Ca	0.02	0.07	0.07	0.19	0.13
Al	0	0	0	0	0.02
Other elements	97.85	98.52	98.88	98.94	98.03

**Table 3:** Chemical analysis of blended fuels (dry basis).

Samples	Industrial Analysis (wt.%)			Element Content(wt.%)						
	A	V	FC	C	H	O	N	S	K	Cl
Fujian bituminous coal	10.01	32.78	57.21	71.60	4.83	11.63	1.08	0.85	0.93	0.25
AW25 wt.%	9.02	42.92	48.06	67.11	5.11	16.62	1.39	0.75	1.34	0.47
AW50 wt.%	6.54	55.68	37.78	60.78	6.17	24.45	1.42	0.64	1.59	0.71
AW75 wt.%	4.48	66.77	28.75	54.26	6.65	32.71	1.47	0.43	1.92	0.96
AW100 wt.%	4.07	76.39	19.72	45.56	6.79	42.11	1.12	0.35	2.22	1.21

**Table 4:** Proximate analysis and ultimate analysis of tested samples.

Samples	Proximate Analysis (wt.%)			Ultimate Analysis (wt.%)				
	A_d_	V_d_	FC_d_	C_d_	H_d_	O_d_	N_d_	S_d_
AW	4.07	76.39	19.72	45.56	6.79	42.11	1.12	0.35

A: ash; V: volatiles; FC: fixed carbon; d: dry basis.

**Table 5:** Analysis of ash composition of AW after combustion at different temperatures.

Ash	Chemical Composition (wt.%)											
	K_2_O	Na_2_O	MgO	Al_2_O_3_	SiO_2_	P_2_O_5_	SO_3_	CaO	TiO_2_	MnO	Fe_2_O_3_	Cl
500 °C	22.54	0.17	18.40	1.24	31.83	2.60	1.54	7.61	0.07	0.30	4.64	10.17
600 °C	21.69	0.18	19.56	1.64	30.62	2.86	1.30	8.00	0.07	0.27	4.90	9.27
700 °C	17.50	0.21	22.94	2.08	32.14	3.38	1.44	9.22	0.08	0.30	4.79	5.94
800 °C	12.80	0.28	25.99	2.31	35.5	4.01	1.81	10.44	0.09	0.33	4.74	0.67
900 °C	11.32	0.24	26.86	2.19	32.96	3.70	2.33	10.21	0.10	0.34	4.91	0.09

**Table 6:** Molar content of AW elements.

Samples	Element Content (kmol/t)													
	C	H	O	N	S	Si	K	Na	Al	Ca	Mg	Fe	Cl	P
AW	37.97	67.90	26.31	0.80	0.11	0.76	0.57	0.09	0.04	0.14	0.10	0.03	0.34	0.05

## Data Availability

All data generated or analysed during this study are included in this published article.
